# Control of gene expression during T cell activation: alternate regulation of mRNA transcription and mRNA stability

**DOI:** 10.1186/1471-2164-6-75

**Published:** 2005-05-20

**Authors:** Chris Cheadle, Jinshui Fan, Yoon S Cho-Chung, Thomas Werner, Jill Ray, Lana Do, Myriam Gorospe, Kevin G Becker

**Affiliations:** 1 Cellular Biochemistry Section, Basic Research Laboratory, Center for Cancer Research, National Cancer Institute, NIH, 9000 Rockville Pike, Bethesda, MD 20892 USA; 2 Laboratory of Cellular and Molecular Biology, National Institute on Aging-Intramural Research Program, NIH, 5600 Nathan Shock Drive, Baltimore MD 21224-6825 USA; 3 Genomatix Software GmbH, Landsberger Str. 6, D-80339 München, Germany; 4 Capital Genomix, 9290 Gaither Road, Gaithersburg, MD 20877 USA; 5 DNA Array Unit, National Institute on Aging-Intramural Research Program, NIH, 5600 Nathan Shock Drive, Baltimore MD 21224-6825 USA

## Abstract

**Background:**

Microarray technology has become highly valuable for identifying complex global changes in gene expression patterns. The effective correlation of observed changes in gene expression with shared transcription regulatory elements remains difficult to demonstrate convincingly. One reason for this difficulty may result from the intricate convergence of both transcriptional and mRNA turnover events which, together, directly influence steady-state mRNA levels.

**Results:**

In order to investigate the relative contribution of gene transcription and changes in mRNA stability regulation to standard analyses of gene expression, we used two distinct microarray methods which individually measure nuclear gene transcription and changes in polyA mRNA gene expression. Gene expression profiles were obtained from both polyA mRNA (whole-cell) and nuclear run-on (newly transcribed) RNA across a time course of one hour following the activation of human Jurkat T cells with PMA plus ionomycin. Comparative analysis revealed that regulation of mRNA stability may account for as much as 50% of all measurements of changes in polyA mRNA in this system, as inferred by the absence of any corresponding regulation of nuclear gene transcription activity for these groups of genes. Genes which displayed dramatic elevations in both mRNA and nuclear run-on RNA were shown to be inhibited by Actinomycin D (ActD) pre-treatment of cells while large numbers of genes regulated only through altered mRNA turnover (both up and down) were ActD-resistant. Consistent patterns across the time course were observed for both transcribed and stability-regulated genes.

**Conclusion:**

We propose that regulation of mRNA stability contributes significantly to the observed changes in gene expression in response to external stimuli, as measured by high throughput systems.

## Background

Virtually all microarray studies to-date have measured changes in steady-state mRNA levels by harvesting total cellular RNA and using it to generate probes through a variety of strategies including end-labeling of purified mRNA [1], incorporating a label into the first strand cDNA made from mRNA [2], or attaching a T7 RNA polymerase promoter during cDNA synthesis, then labeling of the resulting RNA [3]. More recently, several groups have demonstrated the feasibility of hybridizing metabolically labeled mRNAs directly from nuclear run-on (NRO) reactions to nylon filter microarrays in order to investigate nascent transcripts [1,4-6]. Schuhmacher et al. [5], in particular, used a B cell line carrying a conditional, tetracycline-regulated *myc *gene, and found that *myc *induction resulted in only a small overlap in regulated mRNAs at 4 hours post-induction when comparing polyA mRNA and NRO RNA on microarrays. This early work provided evidence that transcriptional activation of genes does not necessarily lead to a corresponding increase of their steady-state mRNA levels.

More recently, our laboratory has examined the relationship between newly transcribed (NRO) RNA and polyA mRNA in a stress model using human non-small lung carcinoma H1299 cells. In response to a variety of stresses (ultraviolet light, heat shock, or prostaglandin), we found that approximately half of the observed changes in mRNA levels of stress-regulated genes were accompanied by a corresponding increase or decrease in gene transcription as measured by NRO. The remaining half of stress-altered changes in gene expression was largely attributable to changes in mRNA turnover, thus suggesting that, on a global level, changes in mRNA turnover profoundly influence gene expression patterns [6].

Several questions, however, remained to be answered from these earlier studies. Since in both the *myc *induction and stress experiments, as mentioned above, measurements of both newly transcribed and polyA mRNAs were made at a single time point, there existed a reasonable possibility of temporal disjunctions between the timing of mRNA new gene synthesis and the rates of accumulation of mRNA in the cell. The second question remaining unanswered is whether or not highly significant levels of mRNA stability regulation (> 50% of all measured gene expression in the stress example) is common to different biological model systems. In order to begin to address these questions we investigated changes at the levels of transcription and total cellular mRNA abundance simultaneously across a time course of activation using Jurkat T cells.

T-cell activation is one of the most widely studied models of cellular response to exogenous stimulation. The initial events include rapid signaling via protein-protein interactions, phosphorylation/dephosphorylation of target signaling molecules, and release of Ca^2+ ^from intracellular stores. Subsequent activation of signal transduction cascades culminates in the implementation of gene expression patterns characteristic of the immune response. Initial microarray studies using T cells have focused on gene expression changes occurring several hours after activation [7-13], even though earlier work using more traditional methods had defined the commitment period for T-cell activation, including alteration in gene expression patterns, as occurring between 1–2 h after exposure to the activating agent [14]. In order to investigate these earlier gene expression changes we chose to examine a time course of activation spanning the first hour after stimulation. While a recent study by Garcia-Martinez et al. [15] in yeast using a similar approach demonstrated large shifts in mRNA stability following a glucose-to-galactose shift, the work presented here is the first systematic accounting of the changes in both gene transcription and mRNA stability in response to a major cellular activation event over a defined time period in higher eukaryotes.

## Results

**Figure 1:**
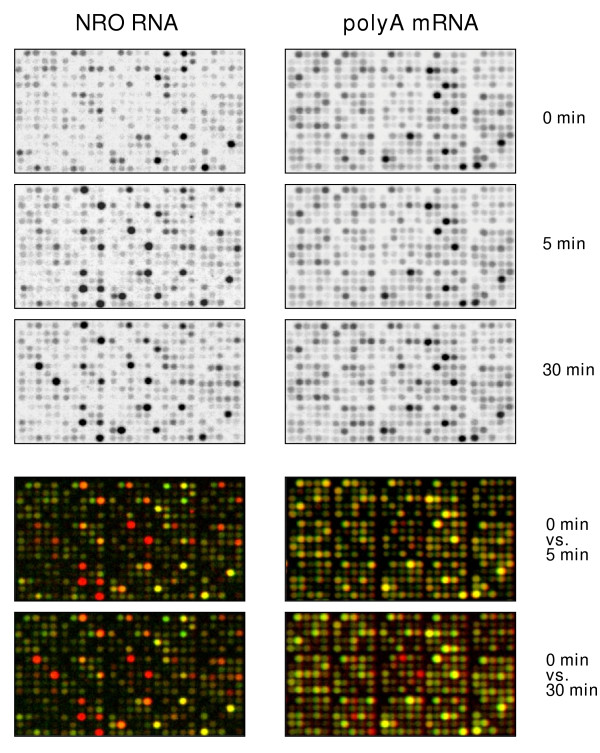
Hybridization images of polyA mRNA and nuclear run-on (NRO) RNA. Depicted are signals in fields of arrays corresponding to untreated (time 0), as well as 5, and 30 min after induction of Jurkat cells using 40 ng/ml PMA and 1 μM Ionomycin (P+I). Two-color overlays contrast either 5 min or 30 min (red) versus 0 (green) for polyA mRNA and NRO RNAs.

T-cell commitment is believed to occur early during activation and therefore changes in gene expression during the earliest stages of induction are of particular interest. An experimental model using human Jurkat T cells activated with PMA and ionomycin was used to investigate early changes in gene expression (up to one hour of stimulation), focusing on both changes in mRNA transcription rates as well as polyA mRNA levels. One hour was chosen in order to examine gene regulatory events occurring immediately after activation and to avoid, for the most part, the influence of secondary gene regulatory mechanisms taking place at later time points (e.g., increased synthesis of transcription factors). NRO RNA was prepared from isolated cell nuclei (Methods) and polyA mRNA from intact cells. Figure 1 shows an example of filter images obtained after hybridization of arrays using either NRO RNA or polyA mRNA. Cells stimulated for 30 minutes exhibited moderate changes in gene expression in the mRNA arrays. In contrast, NRO RNA arrays revealed rapid and robust changes in transcription that were evident as early as 5 minutes following induction. Unexpectedly, a careful analysis of all significant changes in gene expression across the time course revealed that these early-response genes (as identified by NRO) were a relatively small subset of all of the genes shown to be regulated (see below).

**Figure 2:**
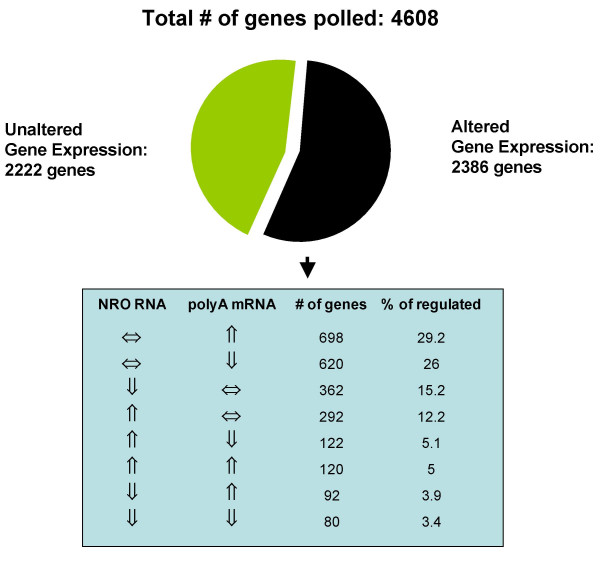
Distributions of significantly regulated genes in both polyA mRNA and nuclear run-on (NRO) RNA. For this analysis, a gene was considered to be up- or down-regulated in either polyA mRNA RNA or NRO RNA (Altered Gene Expression) if it was significantly different from the baseline at any point during the time course of activation; all other genes are in the 'Unaltered Gene Expression' category. The number and per cent of genes in each of 8 possible regulatory categories are displayed.

In all, a total of 4608 genes, including sets of genes enriched for immune response and signal transduction function, were polled (Fig. 2). Of these genes, 2386 showed significant regulation (p < 0.001, or Z ratio > ± 1.5) during the time course of one hour post activation by either changing gene transcription or polyA mRNA levels. These significantly regulated genes were chosen for further analysis.

**Figure 3:**
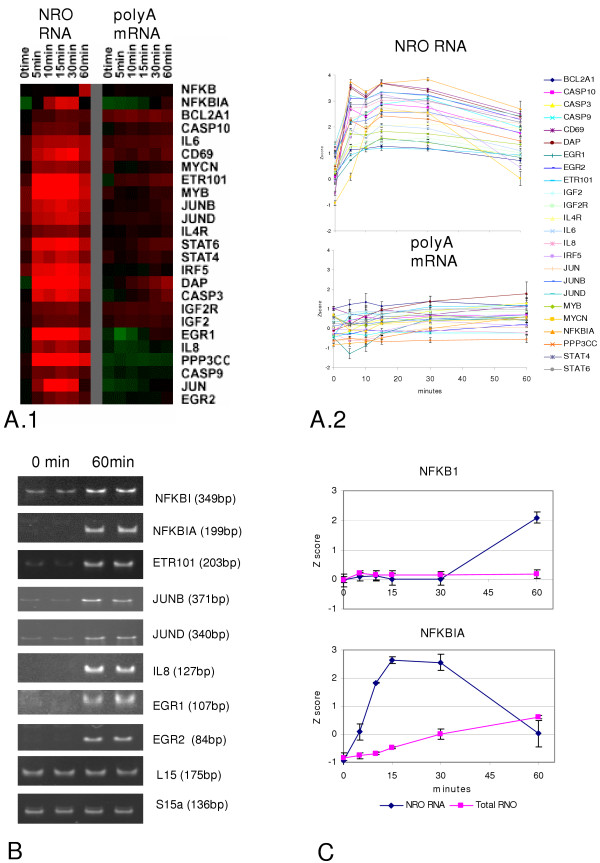
Comparison between polyA mRNA and nuclear run-on RNA of immediate early gene activation in Jurkat T cells. **A**.1 Heatmap of relative gene expression intensities (Z scores). **A.2 **Graphical representation of the same data illustrating an immediately apparent up-regulation of gene expression in NRO but not polyA mRNA. **B**. Single end-point PCR validation of up-regulation in polyA mRNA by 1 hour of a subset of genes shown to be activated within 5 minutes by nuclear run-on RNA. **C**. Patterns of polyA mRNA and nuclear run-on RNA levels for NFKB1 and its inhibitor (NFKBIA).

The distribution and direction (increase, decrease, or no change) of significant changes in gene expression either at the transcriptional level (NRO) or at the level of polyA mRNA (whole-cell) are displayed in the table in Figure 2. The single most common expression event (55.2 %) was an up or down regulation at one or more time points as measured in mRNA without a corresponding (either up or down) regulation as measured by transcription at any time point. The second largest group of regulated genes (27.4%) showed changes in transcription with no corresponding change in polyA mRNA. Examples of genes dramatically up-regulated in this second class included *CD69*, a type II transmembrane receptor involved in lymphocyte proliferation and a classic marker of early T cell activation; *PPP3C*, the catalytic subunit of the calmodulin-activated phosphatase, calcineurin, which plays a central role in signal transduction from the T cell receptor to the nucleus; as well as several members of the *JUN *family of transcription factor immediate early response genes (Fig. 3A).

The final, relatively minor groups of regulated genes included genes which were regulated at both the transcriptional and the polyA mRNA levels in either the same (8.4%) or opposite directions (9%). The relatively low concordance between transcriptional production of mRNA and its measured appearance in polyA mRNA levels was somewhat surprising, although clear examples of coordinated step-wise production were noted for some key genes, as for example, the early response genes *EGR1 *and *ETR101 *(previously shown to be induced at 30 minutes by phorbol ester treatment of a human promyelocytic leukemia cell line [16] and, the apoptosis-related genes *DAP *(death-associated protein, mediator of interferon-gamma-induced apoptosis) and *CASP3*, as well as the immune response signal transducer and activator of transcription (*STAT) 6 *(Fig. 3A).

Dramatic activation of immune response, immediate-early response genes, and apoptosis-related genes was observed in the nuclear run-on RNA as early as 5 minutes following activation (Fig. 3A). This group of genes included immediate-early response genes commonly up-regulated during cellular activation (*ETR101*, *Myb*, *Myc*, and genes of the *JUN *and *EGR *families), genes specifically associated with an early response in immune cells (*IL6*, *IL8*, *STAT4*, *STAT6*, *PPP3C*, *NFKBIA*, *IRF5*, and *CD69*), as well as genes involved in regulating apoptosis (*BCL2A1*, *CASP3*, *CASP9*, *CASP10*, and *DAP*). Many of these same genes were eventually up-regulated in polyA mRNA later in the time course and their increase in expression after one hour was independently validated by single end-point PCR validation using GS320 technology (Fig. 3B).

A particularly interesting example of the dichotomy between transcription and changes in polyA mRNA levels was seen in the production of the mRNAs encoding NFKB1 (NF-kappa B), a key mediator of the transcriptional control of genes involved in the immune response and acute phase reactions, and its inhibitor, NFKBIA (NF-kappa B inhibitor A). Both NFKB1 and NFKBIA have previously been shown by microarray analysis to be significantly induced in polyA mRNA between 3–4 hrs following phorbol or lectin activation of either Jurkat or human peripheral blood lymphocytes [7,8,11,17]. As demonstrated here (Fig. 3C), the production of NFKB1 mRNA clearly increases between 30 minutes and one hour at the transcriptional level without a detectable corresponding increase in polyA mRNA during that time (subsequent PCR analysis did show some increase at the steady-state level between 0 and 60 minutes for the NFKB1 gene but this increase failed to meet the significance thresholds set for the microarray analysis). NFKBIA, on the other hand, is rapidly induced transcriptionally to a maximum level by 30 minutes, returning essentially to baseline within one hour. Meanwhile, NFKBIA steady-state levels can be seen to gradually rise across the first hour of the time course. Analysis of the dynamics of gene expression for NFKB1 and its inhibitor as deduced from conventional microarrays might suggest that by one hour NFKB1 production had not yet begun (contradicted by the NRO data here), and also that the mRNA for the inhibitor of NKFKB1 is steadily increasing (when, in fact, it is clear from NRO data that virtually all increases in the production of NFKBIA have concluded by one hour). These data provide a clear example of how information from nuclear run-on microarrays can enhance studies of gene activation and feedback mechanisms.

**Figure 4:**
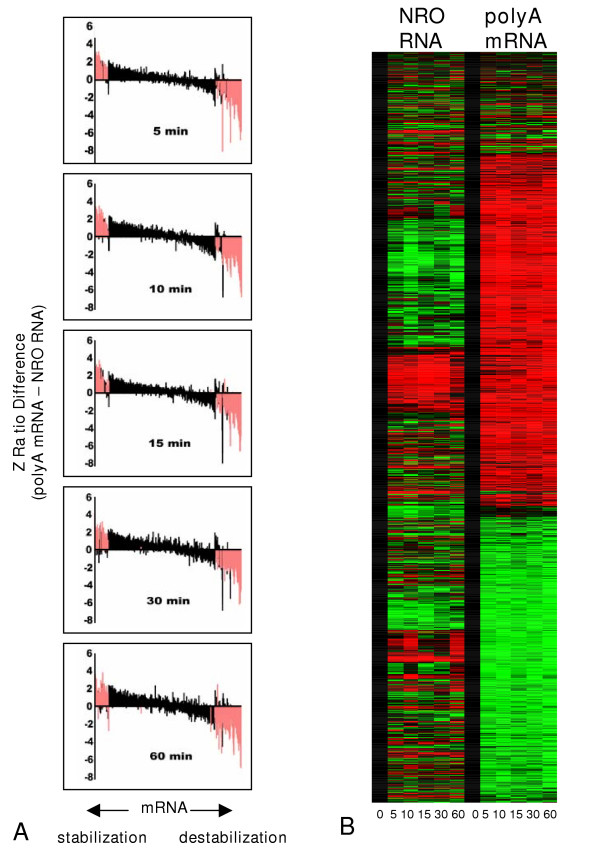
Global comparison of gene expression changes in polyA mRNA and NRO RNA. **A**. At each time period indicated columns correspond to the values derived by subtracting the Z ratio of NRO RNA from the Z ratio of polyA mRNA for every gene. Equivalency between calculations varies around 0, data is aligned using a combined average index, and is displayed from left to right to represent the highest average positive Z ratio difference to the lowest average negative Z ratio difference. Columns in red correspond to genes exhibiting significant differences (Z ratio difference values greater or less than ± 1.5) in gene expression changes when comparing polyA mRNA and NRO RNA for each gene at each time point. **B**. Hierarchical clustering of all significant changes in gene expression in either NRO or polyA mRNA across the time course of activation. The median Z score data for each gene is individually normalized to its baseline value in for the sake of this comparison.

Consistency at each time point for genes regulated by activation-induced changes in mRNA stability can be seen in a graph of the Z ratio differences (Methods) between NRO and polyA mRNA calculated for all genes at all time points (Fig. 4A). These putative stability-regulated genes exhibited a very high degree of consistency at all the time points measured. This replicability is further illustrated in the heat map of clustered gene expression in Figure 4B in which the data for each gene has been independently normalized to its baseline (0 time) level. Large numbers of genes as measured in the polyA mRNA are consistently and strongly regulated following activation. Some of these changes are mirrored by changes in gene transcription (NRO) but most are not.

**Figure 5:**
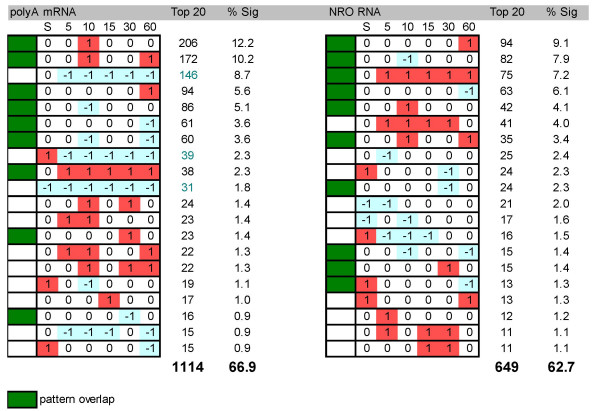
Frequency distribution of gene expression patterns generated from polyA mRNA or NRO RNA. The top 20 patterns for each method is shown. Significant changes in gene expression were assigned a 1, -1, or 0 for up, down, or no change, respectively. In addition, a value of -1, 0, or 1 (low, moderate, or high) was assigned to each gene according to its relative intensity at baseline (0 time). The absolute numbers of genes in each pattern are reported in the column labeled **Top 20 **and the percentage of those genes relative to all significantly regulated genes can be found in the column labeled **% Sig**. Totals are as indicated at the bottom of each column. Filled-in boxes denote patterns equally shared in the top 20 between both methods

**Figure 6:**
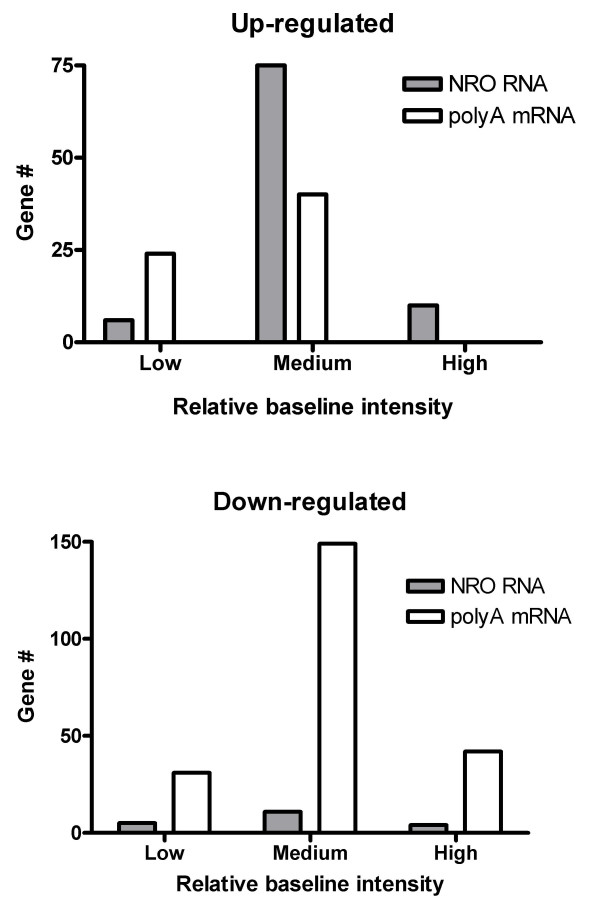
Differential distribution of transcriptional and polyA mRNA up- (A) or down- (B) regulated gene expression patterns during Jurkat T cell activation. The number of genes consistently regulated (up or down at every time point) are correlated with their relative expression baselines (Z score: high>1, 1>medium>-1, low<-1).

In order to compare changes in gene expression patterns at the transcriptional and polyA mRNA levels in a systematic fashion, a simple barcode of 1, -1, or 0 was applied to all significant changes in gene expression indicating up, down, or no change, respectively. In addition, a value of -1, 0, or 1 (low, moderate, or high) was assigned to each gene according to its relative intensity at baseline (0 time). An analysis of the distribution of these gene expression patterns (Fig. 5) revealed that both at the transcriptional and the steady-state levels two thirds of all genes were restricted to just one of 20 patterns (out of a possible number of 729) and that half of these patterns were shared between the two groups. An interesting distinction between the two groups was that whereas up-regulation from a moderately high level of baseline expression was highly favored for new gene synthesis, down-regulation from a moderately high level of baseline expression was very highly favored during polyA mRNA regulation (Fig. 6). In fact, down-regulation, as a consistent trend, was much less common among transcriptional-regulated than steady-state-regulated genes, with implications (see below) as to the roles these modes of regulation play in concert for the control of gene expression.

**Figure 7:**
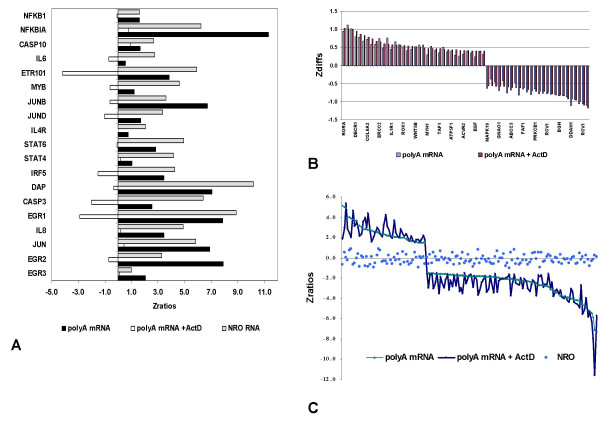
Persistence of stability-regulated changes in gene expression in the presence of Actinomycin D. **A**. Effect of Actinomycin D on the (P+I)-induced changes in the expression patterns of gene deemed to be transcriptionally regulated. Z ratio comparisons are made to the baseline or unactivated state. **B**. Lack of an effect of Actinomycin D on the (P+I)-induced changes in the expression patterns of genes deemed to be stability regulated. The absolute differences (Z diff) between the activated and un-activated state for both polyA mRNA and NRO RNA for a subset of these genes is shown. **C**. Comparison of the Z ratios for all significantly regulated genes in the presence or absence of Actinomycin D showing no corresponding regulation in NRO RNA. Data is sorted by polyA mRNA (without ActD) values and a significance threshold of Z ratios > ± 1.5 was used for these calculations.

In order to confirm that stability regulation was in fact a reasonable explanation for the observation that the expression levels of large numbers of genes were changing at the whole cell but not the transcriptional level, a series of experiments were carried out in which activation of Jurkat cells was carried out in the presence or absence of the transcription inhibitor Actinomycin D. Analysis of polyA mRNA demonstrated the strong inhibition of mRNA levels for genes previously shown to be transcriptionally up-regulated (Fig. 7A). In contrast, large numbers of genes which were significantly regulated in polyA mRNA but not in NRO RNA were not affected by Actinomycin D treatment (Fig. 7B &7C). Among these unaffected genes there was a bias towards presumptively de-stablized genes (Fig. 7C) consistent with the earlier conclusion (Fig. 6B) that down-regulation is the predominant motif in overall polyA mRNA levels.

**Figure 8:**
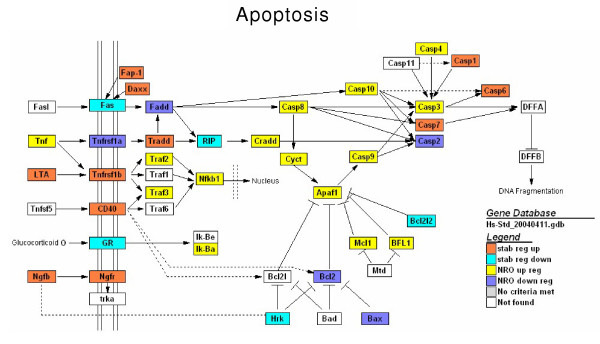
Regulation of apoptotic pathways during T cell activation involves changes in gene expression by both mRNA transcriptional and mRNA stability mechanisms. Genes colored in red and blue were up- or down-regulated in polyA mRNA only. Genes colored in yellow and purple were up- or down-regulated in NRO RNA. Pathway is from a Kegg map modified in Genmapp2.0 [27].

Although an examination of the functional classifications of stability regulated versus transcriptionally regulated genes yielded no obvious trends, some biological pathways appear to be differentially, and sometimes, exhaustively regulated by each type of expression event. One example of this can be seen in the apoptotic pathways, which are comprehensively regulated during the Jurkat activation scenario [16,18,19]. As can be seen from the pathway schematic illustrated in Figure 8, some major effectors of apoptosis such as *CASP 3*, *4*, *8*, *9*, &*10 *(up-regulated) and *BCL2 *(down-regulated), are controlled by new gene synthesis while other factors such as *CASP 1*, *6*, &*7 *(up-regulated) and *BCL2L2 *(down-regulated) appear to be regulated by stability processes alone. Regardless of the cellular rationale for regulating at the level of new gene synthesis or mRNA stability, it is clear from these data that there is a strong internal coherence: genes regulated by one mechanism do not crossover to the other within the time frame investigated.

**Figure 9:**
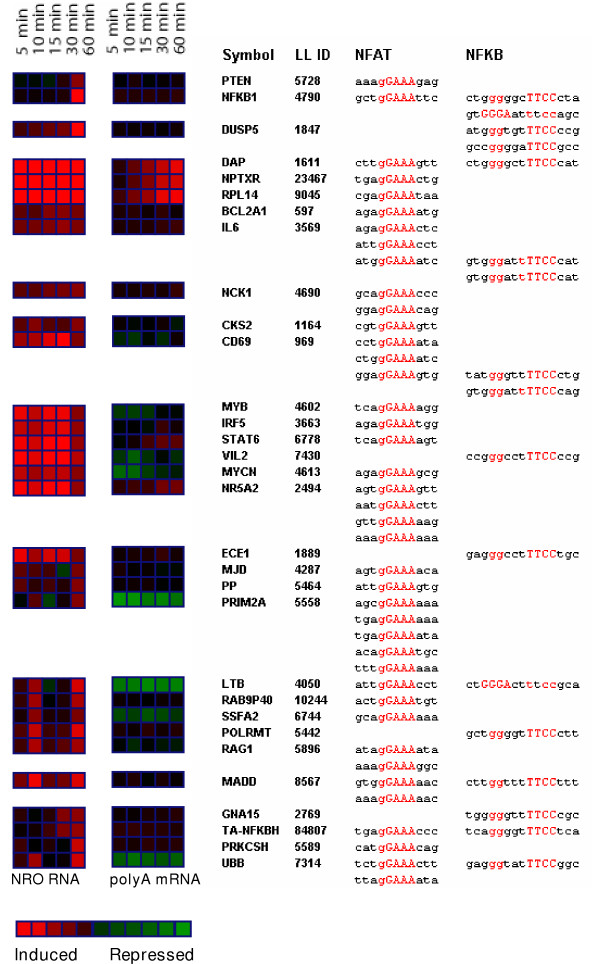
Enrichment of NFAT and NFκB transcription factor-binding sites in the promoter regions of genes upregulated after 60 min of treatment of human Jurkat T cells with PMA + Ionomycin. Color gradient from bright red to bright green is directly proportional to Z ratios and indicates the increase (red) or decrease (green) of gene expression relative to baseline at each of the indicated time points.

Common patterns of transcription factor binding sites in the upstream promoter regions of groups of genes whose transcription was significantly up-regulated were detected. An enrichment of transcription factor-binding sites for both NFAT and NFκB was found in the promoter regions of genes most significantly up-regulated at 1 hour as shown in Figure 9. The frequency with which both NFAT- and NFκB-binding sites were found in the promoters of this group of genes was significantly greater than that seen for other genes in the array [e.g., genes down-regulated at this time point or genes selected at random [supplemental data]. The discovery that NFAT- and NFκB-binding sites were enriched in the promoters of these genes was not unexpected, since these transcription factors, along with AP-1 components Fos and Jun, constitute the major transcription factors involved in the early stages of T-cell activation. Indeed, the frequency of genes significantly up-regulated in NRO RNA and enriched for AP-1 binding sites peaks during the time course (supplemental data). Many of the genes that are transcriptionally upregulated at 1 hour, such as *CD69*, are elevated throughout the time examined, although a few genes including *PTEN*, *DUSP5*, and *NFκB1 *itself, show elevated transcription only after 60 minutes. The simultaneous increase of *NFκB1 *gene transcription (between 30 minutes and 1 hour) combined with a noticeable increase in the transcription of genes containing NFκB-binding sites (at one hour) demonstrates the synchronous relationship between the appearance of this transcription factor and its downstream targets at the indicated time points.

## Discussion

The absence of significant regulation at the transcriptional level during a time in which gene expression is strongly perturbed at the polyA mRNA level during T cell activation reveals that many genes are being regulated through changes in stability. The lack of detectable transcriptional regulation of large numbers of steady-state mRNA gene changes is particularly striking insofar as the NRO measurements are likely to be even more sensitive to changes in gene expression than polyA mRNA measurements since they are a direct measure of newly synthesized mRNA. This finding is consistent with the work of Raghavan et. al. [12,20] who also noted the presence of numerous transcripts exhibiting stimulus-dependent changes in mRNA decay in human T lymphocytes treated for 3 hours with anti-CD3 and/or anti-CD28 antibodies. These experiments were carried out by arresting transcription with Actinomycin D (in the presence or absence of activation), and mRNA turnover rates were globally measured by applying polyA mRNA to microarrays. Similarly, in this current work, patterns of Actinomycin D resistant changes in gene expression among groups of genes significantly regulated only at the polyA mRNA level supports the observation that large numbers of genes are regulated primarily by stability during T cell activation.

The smaller, but still substantial, group of genes which displayed significant transcriptional regulation without any corresponding changes in polyA mRNA, may either be a reflection of the enhanced sensitivity of transcriptional detection or, perhaps, result from a persistent lag between changes in transcriptional output and their reflection in steady-state mRNA levels. Such a lag might arise from differences in scale of the absolute size of mRNA pools between newly transcribed and polyA mRNA. In this scenario, changes in the amounts of mRNA that are readily detectable by nuclear run-on may be too small to have an immediate detectable impact on the steady-state RNA pools, possibly for long periods of time. Another possibility is that the nascent mRNAs of some of these genes are so rapidly degraded that they never significantly impact on polyA mRNA levels at all.

As previously noted (Fig. 6), up-regulation of gene expression was a dominant trend for transcriptionally regulated genes while down-regulation was dramatically favored for genes regulated at the polyA mRNA level. From the standpoint of an overall cellular economy of gene expression, effective control at the level of transcription can be achieved primarily by turning on new gene transcription, while at the whole-cell level, rapid and effective regulation can be achieved by massive shifts in the stability of existing mRNA pools. In fact, the single most dramatic regulatory event experimentally observed was the rapid clearance of large pools of steady-state mRNA, presumably as result of a sudden and demanding change in cellular conditions.

Questions remain as to the extent that this type of regulation may occur under differing biological scenarios. Global regulation of mRNA stability has thus far been systematically studied under conditions of stress and cellular activation. It remains to be determined whether or not changes in mRNA stability are responsible for altering gene expression programs in response to other biological conditions. The existence of these regulatory paradigms may require a reevaluation of the common model of the control of gene expression which essentially invokes the turning on and off of gene transcription in order to explain changes in polyA mRNA levels.

## Conclusion

Although stability regulation might reasonably be considered as a cellular measure to bridge the gap between the very rapid events of signal transduction and the longer term induction of cellular programs involving the coordinated transcription of batteries of new gene synthesis, the current data actually suggests otherwise. One of the goals in this work was to examine changes in gene expression due to altered transcription and those due to altered turnover across a time course allowing sufficient time for the resolution of time disparities between new mRNA synthesis and their appearance as measurable cellular pools. Sufficient time for effective transcription and even translation was clearly demonstrated by the example of NFKB1 mRNA induction followed by the transcription of its downstream targets. Patterns of stability regulated genes were, in this model system, non-random and surprisingly persistent throughout the entire time course suggesting that stability regulation was a major component in the control of gene expression for significant periods of time. The presence of highly active and global regulation of polyA mRNA levels by stability-altering mechanisms suggests a new significant level of regulation in the control of gene expression which spans wide phylogenetic distances from yeast [15] to humans, emphasizes a possible parsimonious role for new gene synthesis in response to changing cellular environments, and may help to explain some of the difficulties encountered in attempts to comprehensively correlate clustered changes in polyA mRNA with common promoter regulatory elements.

## Methods

### Cell culture and treatment

The human Jurkat E6-1 T cell line was cultured in RPMI-1640 medium (GIBCO-BRL, Gaithersburg, MD) supplemented with 10% FCS (Hyclone, Logan, UT) and antibiotics, and incubated at 37°C, 5% CO_2_. Cells were stimulated with 40 ng/ml phorbol 12-myristate 13-acetate (PMA) and 1 μM ionomycin (Sigma, St. Louis, MO) (P+I). Actinomycin D (A.G. Scientific, San Diego, CA) was added at 10 ug/ml 30 minutes prior to activation with PMA + I.

### Preparation and purification of RNA

Isolation of nuclei and preparation of nuclear run-on (NRO) RNA were as described [6,21] with some modifications. Briefly, ~10^8 ^cells per sample were pelleted, washed with PBS, resuspended in ice-cold lysis buffer (20 mM Tris-HCl [pH 7.4], 20 mM NaCl, 5 mM MgCl_2_, 0.25% [v/v] NP-40), and incubated on ice for 5 min. Nuclei were spun down, resuspended in storage buffer (50 mM Tris-HCl [pH 8.3], 5 mM MgCl_2_, 0.1 mM EDTA-NaOH [pH 8.0], 40% [v/v] glycerol) and stored at -80°C until use. Thawed nuclei (200-μl aliquots) were mixed with 200 μl of reaction buffer (5 mM Tris-HCl [pH 8.0], 0.15 mM KCl, 2.5 mM MgCl_2_, 2.5 mM DTT, 0.5 mM of each ATP, UTP, GTP) plus 500 μCi of [α-^33^P]UTP (3,000 Ci/mmol, 10 mCi/ml; ICN), and incubated for 30 min at 30°C with shaking. Samples were then incubated with DNase I (100U, RNase-free; Roche Diagnostics) for 20 min at 37°C, and with proteinase K (1 μg/μl) for 1 h at 37°C. Finally, nascent RNA was purified by Sephadex G-50 column filtration. For the preparation of polyA mRNA cellular RNA, resting or stimulated Jurkat cells were lysed in STAT60™ (TEL-TEST, INC., Friendswood, TX) as described [6]. RNA concentration and quality were assessed spectrophotometrically and by agarose gel electrophoresis. NRO RNA was stored at -80°C until use.

PolyA mRNA samples were radiolabeled and hybridized as previously described [22]. In brief, 5 μg of total mRNA for each sample was annealed, in 16 μl H_2_O, with 1 μg of 24-mer poly(dT) primer (Research Genetics, Alabama), by heating at 65°C for 10 min and cooling on ice for 2 min. The RT reaction was performed by adding 8 μl of 5X first strand RT buffer (Life Technologies, Rockville, MD), 4 μl of 20 mM dNTPs minus dCTP) (Pharmacia, Piscataway, NJ), 4 μl of 0.1 M DTT, 40 U of RNAseOUT (Life Technologies), 6 μl of 3000 Ci/mmol α-^33^P dCTP (ICN Biomedicals, Costa Mesa, CA) to the RNA/primer mixture to a final volume of 40 μl. Two μl (400 U) of Superscript II reverse transcriptase (Life Technologies) was then added, and the sample was incubated for 60 min at 42°C. The reaction was stopped by the addition of five μl of 0.5 M EDTA. The samples were incubated at 65°C for 30 min after addition of 10 μl of 0.1 M NaOH in order to hydrolyze and remove RNA. The samples were pH neutralized by the addition of 25 μl of 0.5 M Tris, pH 8.0, and purified using Bio-Rad 6 purification columns (Hercules, CA). An aliquout of each labled sample was quantitated by liquid scintillation counting using a Beckman LS 6500. The entire remaining sample was stored at -20°C until use.

### Array construction and hybridization

Microarray construction and hybridization were previously described [23]. Briefly, NIA Human Focused Arrays consisting of a set of 4600 spotted cDNAs, arrayed in duplicate, representing a set of 2742 non-redundant genes (enriched in genes involved in immune function and signal transduction), were printed on Nytran + Supercharge nylon membranes (Schleicher & Schuell, Keene, NH), and hybridized with [α-^33^P]dCTP-labeled cDNA or [α-^33^P]UTP RNA probes overnight at 50°C as previously described [22], protocols available at . Hybridized arrays were rinsed in 2 X SSC and 0.1% SDS twice at 55°C followed by washes in 2 X SSC and 0.1% SDS at 55°C. Microarrays were exposed for 1–3 d and scanned using a PhosphorImager (Molecular Dynamics, Sunnyvale, CA) at a 50-μm pixel resolution. ArrayPro software (MediaCybernetics, Silver Spring, MD) was used to convert the hybridization signals into raw intensity values; data generated were transferred into Microsoft Excel for further analysis.

### PCR analysis

Single endpoint PCR was carried out on polyA mRNA for gene detection and relative quantitative comparison between baseline (0 time) and I hour activation (with PMA +I). In brief, GS320 libraries for both control and activated polyA mRNA were prepared as described [24], normalized using a panel of ribosomal protein genes, and specific genes were amplified in duplicate using predefined GS320 primers.

### Array data analysis

RNA samples (typically n = 3 to n = 5) were prepared from multiple experiments, each of which consisted of a consecutive series of time points. Equal amounts of total RNA (5 μg) or NRO RNA (10^8 ^cell-equivalents) were used in each hybridization. Raw intensity data for each experiment was transformed to log_10_, then used for the calculation of Z scores as described [22] [see Additional file 1]. Significant changes in gene expression were calculated in the form of Z ratios and/or Z test values ([25]), using Z score values in all calculations. Z ratios constitute a measure of the change in gene expression of a given gene from its baseline value (in this case – time 0), expressed in units of standard deviation from the average change of all genes for that comparison. Z ratios are a direct measure of the likelihood that an observed change is an outlier in an otherwise normal distribution and, as such, are independent from underlying intensity values. Since the contents of the population of nuclear run-on and polyA mRNA are different in both complexity and number, care was taken not to compare Z score normalized intensities directly. Comparisons between Z ratios, however, test for equivalence of significant changes between the transcriptional and steady-state changes in gene expression each relative to its own population. All gene expression changes were assessed through comparison with untreated cells (time 0). A Z ratio value of ± 1.50 and/or a Z test value p < 0.0001 were the significance thresholds used in this study.

Hierarchical clustering was performed using the Cluster and TreeView software programs, developed at Stanford University [26]. The clustering algorithm was set to complete linkage clustering using the uncentered Pearson correlation.

Mapping of transcription factor binding sites in selected genes was performed using software from Genomatix Software Gmbh, Munich, Germany . See Additional file 2 for a complete description of the analysis.

## Abbreviations

NRO, nuclear run-on; P+I, PMA plus Ionomycin; ActD, Actinomycin D

## Authors' contributions

CC and JF performed the microarray assays (JF-NRO RNA, CC-polyA mRNA). CC carried out statistical analysis, and drafted the manuscript (with MG & KGB). JR and LD performed the PCR validation assays. TW assisted with promoter analysis and YSC-C participated in the design of the study. All authors read and approved the final manuscript.

## Supplementary Material

Additional File 1 Cheadle et al – BMC Genomics – basic data 05-27-05.xls, contains all original data (normalized), significance testing, and barcode assignments. Click here for file

Additional File 2 Cheadle et al – BMC Genomics TF binding sites frequency analysis 05-27-05.xls, contains all calculations for TF binding site analysis. Click here for file

## References

[B1] Legen J, Kemp S, Krause K, Profanter B, Herrmann RG, Maier RM (2002). Comparative analysis of plastid transcription profiles of entire plastid chromosomes from tobacco attributed to wild-type and PEP- deficient transcription machineries. Plant J.

[B2] Schena M, Shalon D, Davis RW, Brown PO (1995). Quantitative monitoring of gene expression patterns with a complementary DNA microarray. Science.

[B3] Eberwine J (1996). Amplification of mRNA populations using aRNA generated from immobilized oligo(dT)-T7 primed cDNA. Biotechniques.

[B4] Meininghaus M, Chapman RD, Horndasch M, Eick D (2000). Conditional expression of RNA polymerase II in mammalian cells. Deletion of the carboxyl-terminal domain of the large subunit affects early steps in transcription. J Biol Chem.

[B5] Schuhmacher M, Kohlhuber F, Holzel M, Kaiser C, Burtscher H, Jarsch M, Bornkamm GW, Laux G, Polack A, Weidle UH, Eick D (2001). The transcriptional program of a human B cell line in response to Myc. Nucleic Acids Res.

[B6] Fan J, Yang X, Wang W, Wood WH, Becker KG, Gorospe M (2002). Global analysis of stress-regulated mRNA turnover by using cDNA arrays. Proc Natl Acad Sci U S A.

[B7] Ellisen LW, Palmer RE, Maki RG, Truong VB, Tamayo P, Oliner JD, Haber DA (2001). Cascades of transcriptional induction during human lymphocyte activation. Eur J Cell Biol.

[B8] Cristillo AD, Bierer BE (2002). Identification of novel targets of immunosuppressive agents by cDNA- based microarray analysis. J Biol Chem.

[B9] Marrack P, Mitchell T, Hildeman D, Kedl R, Teague TK, Bender J, Rees W, Schaefer BC, Kappler J (2000). Genomic-scale analysis of gene expression in resting and activated T cells. Curr Opin Immunol.

[B10] Rogge L, Bianchi E, Biffi M, Bono E, Chang SY, Alexander H, Santini C, Ferrari G, Sinigaglia L, Seiler M, Neeb M, Mous J, Sinigaglia F, Certa U (2000). Transcript imaging of the development of human T helper cells using oligonucleotide arrays. Nat Genet.

[B11] Feske S, Giltnane J, Dolmetsch R, Staudt LM, Rao A (2001). Gene regulation mediated by calcium signals in T lymphocytes. Nat Immunol.

[B12] Raghavan A, Ogilvie RL, Reilly C, Abelson ML, Raghavan S, Vasdewani J, Krathwohl M, Bohjanen PR (2002). Genome-wide analysis of mRNA decay in resting and activated primary human T lymphocytes. Nucleic Acids Res.

[B13] Grolleau A, Bowman J, Pradet-Balade B, Puravs E, Hanash S, Garcia-Sanz JA, Beretta L (2002). Global and specific translational control by rapamycin in T cells uncovered by microarrays and proteomics. J Biol Chem.

[B14] Crabtree GR (1989). Contingent genetic regulatory events in T lymphocyte activation. Science.

[B15] Garcia-Martinez J, Aranda A, Perez-Ortin JE (2004). Genomic run-on evaluates transcription rates for all yeast genes and identifies gene regulatory mechanisms. Mol Cell.

[B16] Shimizu N, Ohta M, Fujiwara C, Sagara J, Mochizuki N, Oda T, Utiyama H (1991). Expression of a novel immediate early gene during 12-O-tetradecanoylphorbol-13-acetate-induced macrophagic differentiation of HL-60 cells. J Biol Chem.

[B17] Schena M, Shalon D, Heller R, Chai A, Brown PO, Davis RW (1996). Parallel human genome analysis: microarray-based expression monitoring of 1000 genes. Proc Natl Acad Sci U S A.

[B18] Kizaki H, Tadakuma T, Odaka C, Muramatsu J, Ishimura Y (1989). Activation of a suicide process of thymocytes through DNA fragmentation by calcium ionophores and phorbol esters. J Immunol.

[B19] Ruiz-Ruiz MC, Oliver FJ, Izquierdo M, Lopez-Rivas A (1995). Activation-induced apoptosis in Jurkat cells through a myc-independent mechanism. Mol Immunol.

[B20] Raghavan A, Bohjanen PR (2004). Microarray-based analyses of mRNA decay in the regulation of mammalian gene expression. Brief Funct Genomic Proteomic.

[B21] Gorospe M, Wang X, Holbrook NJ (1998). p53-dependent elevation of p21Waf1 expression by UV light is mediated through mRNA stabilization and involves a vanadate-sensitive regulatory system. Mol Cell Biol.

[B22] Cheadle C, Vawter M, Freed WJ, Becker KG (2003). Analysis of microarray data using Z score transformation. Journal of Molecular Diagnostics.

[B23] Barrett T, Cheadle C, Wood WB, Teichberg D, Donovan DM, Freed WJ, Becker KG, Vawter MP (2001). Assembly and use of a broadly applicable neural cDNA microarray. Restor Neurol Neurosci.

[B24] Wang A, Pierce A, Judson-Kremer K, Gaddis S, Aldaz CM, Johnson DG, MacLeod MC (1999). Rapid analysis of gene expression (RAGE) facilitates universal expression profiling. Nucleic Acids Res.

[B25] Nadon R, Woody E, Shi P, Rghei N, Hubschle H, Susko E, Ramm P, Geschwind D and Gregg J (2002). Statistical inference in array genomics.. Microarrays for the Neurosciences.

[B26] Eisen MB, Spellman PT, Brown PO, Botstein D (1998). Cluster analysis and display of genome-wide expression patterns. Proc Natl Acad Sci U S A.

[B27] Dahlquist KD, Salomonis N, Vranizan K, Lawlor SC, Conklin BR (2002). GenMAPP, a new tool for viewing and analyzing microarray data on biological pathways. Nat Genet.

